# Tissue/Biofluid Specific Molecular Cartography of *Leishmania donovani* Infected BALB/c Mice: Deciphering Systemic Reprogramming

**DOI:** 10.3389/fcimb.2021.694470

**Published:** 2021-07-29

**Authors:** Sanchita Das, Tanaya Saha, Chandrima Shaha

**Affiliations:** Cell Death and Differentiation Laboratory, National Institute of Immunology, New Delhi, India

**Keywords:** *Leishmania*, metabolomics, GC–MS, host–pathogen interaction, tissue, biofluids, biomarkers

## Abstract

Pathophysiology of visceral leishmaniasis (VL) is not fully understood and it has been widely accepted that the parasitic components and host immune response both contribute to the perpetuation of the disease. Host alterations during leishmaniasis is a feebly touched area that needs to be explored more to better understand the VL prognosis and diagnosis, which are vital to reduce mortality and post-infection sequelae. To address this, we performed untargeted metabolomics of *Leishmania donovani* (Ld) infected, uninfected and treated BALB/c mice’s tissues and biofluids to elucidate the host metabolome changes using gas chromatography–mass spectrometry. Univariate and multivariate data treatments provided numerous significant differential hits in several tissues like the brain, liver, spleen and bone marrow. Differential modulations were also observed in serum, urine and fecal samples of Ld-infected mice, which could be further targeted for biomarker and diagnostic validations. Several metabolic pathways were found to be upregulated/downregulated in infected (TCA, glycolysis, fatty acids, purine and pyrimidine, etcetera) and treated (arginine, fumaric acid, orotic acid, choline succinate, etcetera) samples. Results also illustrated several metabolites with different pattern of modulations in control, infected and treated samples as well as in different tissues/biofluids; for e.g. glutamic acid identified in the serum samples of infected mice. Identified metabolites include a range of amino acids, saccharides, energy-related molecules, etcetera. Furthermore, potential biomarkers have been identified in various tissues—arginine and fumaric acid in brain, choline in liver, 9-(10) EpOME in spleen and bone marrow, N-acetyl putrescine in bone marrow, etcetera. Among biofluids, glutamic acid in serum, hydrazine and deoxyribose in urine and 3-Methyl-2-oxo pentanoic acid in feces are some of the potential biomarkers identified. These metabolites could be further looked into for their role in disease complexity or as a prognostic marker. The presented profiling approach allowed us to attain a metabolic portrait of the individual tissue/biofluid modulations during VL in the host and represent a valuable system readout for further studies. Our outcomes provide an improved understanding of perturbations of the host metabolome interface during VL, including identification of many possible potential diagnostic and therapeutic targets.

## Introduction

Visceral leishmaniasis (VL) is a systemic disease that shows varied clinical manifestations and homing of the pathogen i.e. *Leishmania* to different visceral organs like liver and spleen further affecting bone marrow and brain (Melo et al., 2017; Burza et al., 2018). The interaction of *Leishmania* pathogens with mammalian hosts leads to a variety of physiological responses like fever, malaise, weight loss and anaemia (Walker et al., 2014). Apart from this, engorgement of the spleen, liver and lymph nodes are also observed (Varma and Naseem, 2010). On a cellular level, these responses are comprised of multiple metabolic changes in the affected host cells which are most obvious when the pathogen replicates within host cells, thus, making the metabolome the closest correlation (Brandt et al., 2020). How the parasite modulates the host cell metabolism remains poorly understood. Current therapeutic options consist of anti-inflammatory medication as well as combination therapy; however, several individuals fail to respond to these therapies as they are associated with numerous side effects (Moore and Lockwood, 2010; Ponte-Sucre et al., 2017). Studies for the betterment of therapeutic interventions have been explored widely but the diagnostic sector remains deficient (Lévêque et al., 2020). Present diagnostic methods include invasive techniques; where splenic biopsies and bone marrow aspirates analysis remain the method of choice to date (although it is performed by expert medical practitioners only) (Pastorino et al., 2002; Joob and Wiwanitkit, 2017). Therefore, less invasive methods for diagnosis such as the determination of biomarkers from urine, serum or feces would be more advantageous for early diagnosis. The determination of specific metabolites in the biofluids through metabolic profiling is one of the interesting areas that need to be explored. Furthermore, detection of differential or unique metabolites in various tissues could provide information to explain the pathophysiological stage of VL and can be even more useful in dealing with relapses. This method would be more beneficial as well as less expensive in diagnosing and treating VL.

Metabolomics is one of the latest systems biology approaches for the identification of complete metabolic profile alterations of endogenous substances in biological systems. It aims to identify and quantify all metabolites present in a specific biological sample, followed by its characterization and classification, that help in identifying biomarker(s) patterns; indicative of the physiological state of disease. This is an established approach in clinical infection studies and parasitology (Pacchiarotta et al., 2012; Hocher and Adamski, 2017; Guasch-Ferre et al., 2018). Earlier studies have focused on cellular modifications *in vitro*, but it is still lagging in the *in vivo* assessment, so as to address the detailed trend of composition and heterogeneity of host during VL (Lamour et al., 2012). This study was undertaken in order to decipher the tissue/biofluid specific metabolic changes that occurs during *Leishmania* infection. Hence, the *in vivo* profiling study done here will identify hits that will be more reliable for pinpointing biomarkers.

## Materials and Methods

### Reagents, Cell and Parasite Culture

M199, FBS, Molecular grade water, methanol, chloroform, ribitol (adonitol), methoxyamine HCl, pyridine and primers from Sigma-Aldrich, St. Louis, MO, USA. BSTFA was obtained from Supelco Sigma, Bellefonte, Pennsylvania, United States. SYBR™ green PCR master mix was obtained from Applied Biosystems Foster City California, United States. Ketamax 50 was obtained from Troikaa Pharmaceuticals Ahmedabad, Gujrat, India. DNeasy blood and tissue kit was provided by Qiagen Hilden, Germany. A strain of *L. donovani*, BHU1260, was derived from the splenic aspirate of a VL patient at the Kala Azar Medical Centre of the Institute of Medical Sciences, Banaras Hindu University, Varanasi, India. *L. donovani* strain was grown in M199 medium (pH 7.4), which was supplemented with 10% fetal bovine serum (FBS) (Gibco, Carlsbad, CA, USA) and maintained at 23°C.

### Animal Ethics

All animal experiments were duly approved by the Animal Ethics Committee of the National Institute of Immunology, New Delhi (IAEC/AQ/2019/185, serial no. IAEC#454/17).

### Maintaining Infection in Animals

For *in vivo* experiments, 6 to 10 weeks inbred BALB/c mice were used. They were grouped as control and infected. In the infected group, stationary phase *Leishmania* promastigotes were introduced through intra-cardiac injection with 50 µl 1× PBS containing 1 × 10^6^ parasites on day 0 of the study (Loeuillet et al., 2016). On day 14, the same formulation was used to give intra-peritoneal injection. At the end of day 21, the infected group was sacrificed. Another group was made from the infected BALB/c mice that were treated with miltefosine after the infection was maintained, i.e. on day 21 of infection. Five daily doses of 3 mg/kg was given orally (Dorlo et al., 2012). At the end of day 26, the treated mice were sacrificed.

### Parasitic Load Determination

Detection of *L. donovani* DNA in mouse tissue was carried as described elsewhere (Nicolas et al., 2002). For this, DNA was isolated from the tissues (25 mg) using DNeasy tissue kit. The following forward and reverse primers, 5’CCTATTTTACACCAACCCCCAGT-3 and 5’-GGGTAGGGGCGTTC TGCGAAA-3’ respectively, were used to amplify a 120-bp fragment of the minicircle kDNA of *L. donovani*; 10,000 copies of which are present in each parasite. These primers match the conserved sequences of the kinetoplast minicircle but do not match the mouse frequent nucleic acid sequences. A real-time hot-start PCR was performed with the LC FastStart DNA Master SYBR Green I Kit (Roche Diagnostics, Meylan, France) in an LC (Roche Diagnostics). The 12 µl reaction mixture contained 1 µl LC FastStart DNA Master SYBR Green I, 2 mM MgCl2, 10 µM each of the primers, and 100 ng of the template. Time and temperatures for PCR configuration were as follows: denaturation was done for 8 s at 95°C, amplification of 40 cycles was done at 95°C for 10 s, followed by 72°C for 8 s. The melting cycle was set at 95°C for 10 s, 67°C for 30 s and 95°C for 10 s followed by cooling at 40°C for 60 s. Slope (°C/s) was kept constant at 20 except in the last step of melting where the slope was 0.1. For fluorescence signal acquisition, channel F1 was used and the gain was set at 5. For normalization of fluorescent data, the F1/1 ratio was applied.

### Sample Collection and Preparation

#### Sample Collection

Mice were sacrificed on the 21st day along with the control mice for brain, liver, spleen and bone marrow tissues. Blood (200 µl) from mice was collected just before the sacrifice for serum. Urine and feces were collected on the 21st day of infection (in the morning, afternoon and evening before sacrifice) along with that of the control mice. The treated mice were sacrificed on the 27th day (after following 5 days of treatment). The blood, tissues and other biofluids were collected in a similar manner as described for control and infected mice.

#### Sample Preparation

Sample preparation of brain (Hu et al., 2012), bone marrow (Connor et al., 2018) was done as per the reference protocol. Whereas liver, spleen, serum, urine and feces was done as described elsewhere (Xiao et al., 2016; Jain et al., 2019). In brief, 40 mg of a tissue sample (liver, spleen, bone marrow and feces) was homogenized with water, methanol, chloroform in the ratio of 2:5:2 respectively for a total volume of 1 ml. It was then centrifuged to acquire a bi-phasic solution—aqueous and organic, which were then separated and dried under nitrogen gas for GC derivatization. For brain samples, whole brain was isolated to minimize the heterogeneity of sample, washed with 1× PBS and snap freezed in liquid nitrogen followed by homogenization in water (2 ml/gm). Required volume (volume containing 40 mg of brain tissue) is taken for methanol chloroform extraction. Rest of the protocol was same as that of liver sample preparation. For serum and urine sample preparation, 40 µl of serum/urine was taken to which 150 µl of methanol–chloroform was added in the ratio of 3:1 respectively. The sample was then centrifuged, and the supernatant was dried under nitrogen gas for GC derivatization. For derivatization of samples, 40 µl Methoxyamine HCl (20 mg/ml dissolved in pyridine) was added to the sample and incubated at 37°C for 90 min, followed by addition of 50 µl BSTFA and incubation at 70°C for 60 min. The final volume was made up to 250 µl with pyridine for GC–MS sample processing (Pan et al., 2010).

### GC–MS

Gas–Chromatography was performed with GCMS-QP2010 Ultra (Make—Shimadzu) using Rxi-5 siloxane ms (dimensions: 30 m × 0.25 mm 1 d × 0.25 µm) column for separation. MS conditions were as follows: Ion source temperature was 230.0°C, interface temperature was 270.0°C, solvent cut time was 6.50 min, detector gain mode was kept relative, +0.00 kV was gained by detector whereas the threshold was 1,000. The program start time was 7 min and the end time was 50 min. The acquisition mode was set to scan mode. The event time was 0.20 s and the scan speed was 3,333. The m/z start value was 40 whereas the end value was 650.00.

### Data Analysis With MetaboAnalyst

All GC–MS raw data files were first converted to CDF format with the help of GC–MS post run analysis software which is complementary to the instrument for basic processing of data. These files were then individually uploaded and analyzed in XCMS Online software (www.xcmsonline.scripps.edu). Within the software, a single job was created using the GC/single quad centwave setting where the polarity was set to negative. After the job was completed, the analysed result was available for download in a zip folder format. The following data columns—mass/charge (‘mz’), retention time ‘rt’ and original intensity (‘into’) were copied from the provided excel sheet and saved in a.txt file, which was then converted to a.csv file for further processing and analysis of data in the MetaboAnalyst software (metaboanalyst.ca/faces/home.xhtml). MetaboAnalyst schematics include data normalization, followed by statistical analysis, ‘peak to pathway’ and enrichment analysis (Chong et al., 2018).

### Peak Alignment and Data Normalization

For peak picking and alignment, mass tolerance (m/z) was set to 0.25 and retention time tolerance to 5 s. Peaks of the same group were summed if they belonged to one sample. Peaks appearing in less than half of all samples in each group were ignored. The aligned peaks were reorganized into a single data matrix with the samples in rows and the variables (peaks) in columns for further analysis. Data normalization was done to reduce any systematic bias and to improve overall data consistency so that meaningful biological comparisons can be made. A combination of row-wise normalization by a pooled or average sample of the control group and auto data scaling was used for data normalization. This was observed to be the most suitable method that helped achieve a Gaussian curve, required for further analysis (Chong et al., 2018).

### Statistical Analysis

T-test/ANOVA was employed for 2-group/3-group statistical analyses respectively, as the data sets tend to follow a normal distribution and could possibly have unknown variances. Important features were selected by setting the threshold to 0.05. The absolute value changes between both groups are compared with fold change, therefore, the data before column normalization was used instead. Important features were selected by fold-change analysis with threshold ≥2. Statistical significance (P-value) *versus* magnitude of change (fold change) is represented by a volcano plot, which is essentially a variant of the scatter plot. Features having a fold change threshold of 2 (x-axis) and t-test threshold of (y-axis) 0.1 were considered for further analysis (Chong et al., 2018).

### Pathway Analysis and Enrichment

Using the peaks to pathways module in MetaboAnalyst, we were able to identify significant metabolites. The algorithm type was set to Metabolite Set Enrichment Analysis (MSEA) and the used pathway library was *Mus musculus* (KEGG). The significant metabolites identified were then proceeded for enrichment analysis using Over Representation Analysis (ORA), done in reference to HMDB using the Small Molecule Pathway Database (SMPDB). The fold enrichment factor of a pathway used here for comparison was calculated as the ratio between the number of significant pathway hits and the expected number of compound hits within the pathway (Chong et al., 2018).

### Pattern Hunter

Direction targeting Spearman rank correlation was used as a distance measure, that calculates the ranks of individual features rather than their actual values. A predefined profile of 1-2-1 was used to target features that showed anomaly during infection and remained more or less constant in control and treated samples. For this, all three groups of datasets—control, infected and treated samples were used for comparison (Chong et al., 2018).

## Results

**Figure 1:**
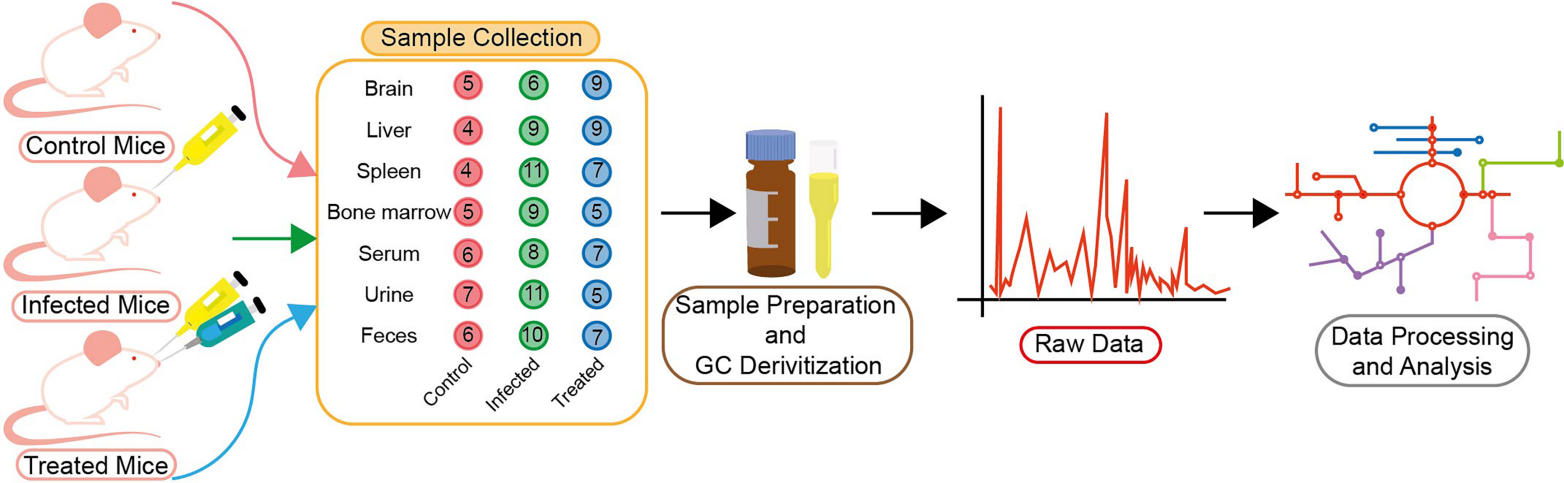
Schematic of experimental plan. BALB/c mice were divided into three groups—control (red), infected (green) and treated (blue). The number of BALB/c mice used for sample collection in control, infected and treated groups is shown in the figure. Tissue and biofluid samples were collected from each group (the digits in the circle designate the number of mice) as shown. Samples were then prepared for GC derivatization followed by their processing in GC–MS (GCMS-QP2010 Ultra). Total Ion Chromatogram (TIC) was acquired here, and was further utilized for data processing and analysis.

**Figure 2:**
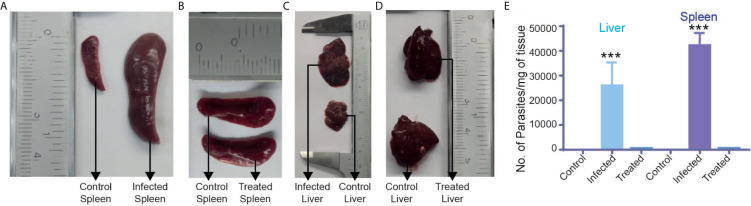
Parasite load determination: Representative images of **(A)** control and infected spleens showing enlargement in the infected spleen. **(B)** Control and treated spleens showing normalization of the spleen after treatment with miltefosine. **(C)** Control and infected liver showing enlargement in the infected liver. **(D)** Control and treated liver showing normalization of the liver after treatment. **(E)** Parasitic load determination in control, infected and treated liver and spleen samples using RT-PCR. ***p ≤ 0.001.

The study reported here is mainly concerned with the identification of BALB/c metabolic alterations during *Leishmania* infection in several tissues like the brain, liver, spleen, bone marrow and biofluids like urine, serum and feces (**Figure 1**). Mice that had been infected successfully were first identified by enlargement of spleen and liver and then proceeded for parasite load determination. Only samples that had a significant parasite load as compared to control ones, were considered to be infected samples as identified through RT-PCR (**Figure 2**).

### Univariate and Multivariate Analyses

**Figure 3:**
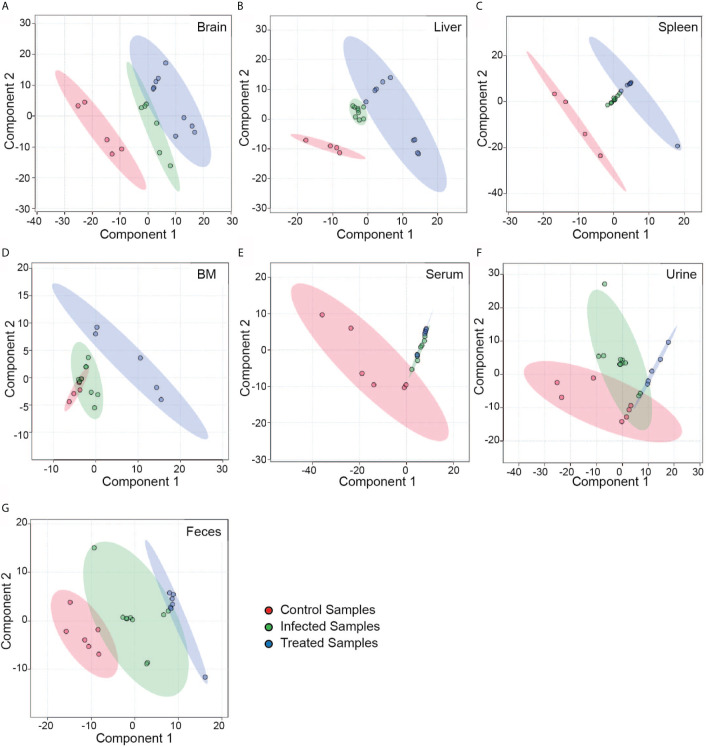
Partial Least Square Determinant Analysis (PLS-DA). A supervised classification method that uses group labels to maximize the separation between groups. Here, we have shown the separation of control (red), infected (green) and treated (blue) groups in different tissue and biofluid samples using a 2D score plot of the first two components. Separation of the different groups are represented in a 2D score plot with 95% confidence ellipse regions based on the data points for individual groups in different tissues—**(A)** Brain, **(B)** Liver, **(C)** Spleen, **(D)**; Bone marrow and biofluids—**(E)** Serum, **(F)** Urine, and **(G)** Feces. In **(D)** and **(E)** the overlap seen among the groups are on different spatial planes as visualized in 3D score plot (data not shown).

To obtain a data overview, multivariate analysis was done to acquire a principal component analysis (PCA) plot, where the correlation among the datasets was obtained through a two-dimensional (2D) graph (**Figures 3A–G**). The 2D score plot draws a 95% confidence region for each group. The principal components were scaled to view separation among the control, infected and treated datasets of different tissue and biofluid samples. Results showed significant variations among the groups, which was clearly visible even in different tissue types and biofluids. A stark difference between the peak intensity patterns of the control, infected and treated groups was evident from the heat map (**Figures S1A–G**), further corroborating the PLS-DA (**Figure 3**) and suggesting that these are indeed three separate groups with significant variations.

In case of brain samples, a fold change analysis indicated that infected samples were downregulated in majority of the features, and the degree of downregulation was much higher than that of the upregulated features in comparison to control samples. A total of 145 significant features were identified for the brain samples *via* volcano plot, where 88 features were upregulated and 91 features were downregulated (**Figure S2A**), whereas, in treated brain samples two upregulated and seven downregulated features were noticed as compared to control samples (**Figure S3A**). The downregulation of the features in infected brain samples was also noticeable in the heatmap (**Figure S1A**). More than 100 significant features were identified in liver samples. A clear demarcation was also visible in liver samples (**Figure 3B**), where a total of 102 upregulated and 10 downregulated significant features were identified while comparing infected *vs* control samples (**Figure S2B**). On the other hand, treated liver samples showed only four upregulated and 11 downregulated features in the volcano plot (**Figure S3B**). Infected splenic samples showed higher intensity features as compared to the control ones; the difference is discernable in the heat map (**Figure S1C**). A little more than 100 significant features were identified that had a threshold greater than 0.05. Majority of the infected group features seem to be upregulated with respect to the control groups in splenic samples, where 62 upregulated features and 47 downregulated features were identified (**Figure S2C**). A similar pattern was also visible in treated splenic samples where 13 downregulated features and a single upregulated feature were identified against control (**Figure S3C**). A clear separation of all the groups was observed with 2D (**Figure 3D**, **Figure S1D**) and 3D pls-da (figure not shown) in bone marrow samples. Three downregulated and seven upregulated features were identified on comparison of control and infected samples (**Figure S2D**). In the case of control *vs* treated bone marrow samples, two upregulated and five downregulated features were observed (**Figure S3D**).

Metabolic alterations were noticeable in the case of biofluids, where all of them (serum, urine and feces) showed differential and significant features. On comparison of control and infected groups, seven significant features were identified with the help of a t-test as the overall peak groups and individual features identified in serum samples were significantly less. Approximately 22 features were found to be upregulated while 18 were downregulated in the volcano plot (**Figure S2E**). Whereas, among the control *vs* treated group only six downregulated and four upregulated features were identified against the control ones (**Figure S3E**). In urine samples, fold change analysis suggested that the features from the infected samples were upregulated more than the control samples (**Figure S1F**). Less than 70 significant features were identified with the statistical t-test analysis. The volcano plot showed 83 upregulated and 44 downregulated features as compared to control (**Figure S2F**). Whereas in the treated *vs* control urine samples only a single downregulated feature was identified (**Figure S3F**). In fecal samples, two completely different sets of patterns seem to emerge from the control and infected groups (**Figure S1G**). Approximately 72 features were identified to be significant with t-test. The volcano plot shows that approximately 29 features were upregulated whereas 68 features were downregulated suggesting that majority of the significant features selected were downregulated (**Figure S2G**). The fecal samples of the treated groups showed all six significant features in the downregulated category (**Figures S3G**).

### Identifying VL Development Through Tissue/Biofluids Specific Metabolic Pathways Alterations During *Leishmania* Infection

**Figure 4:**
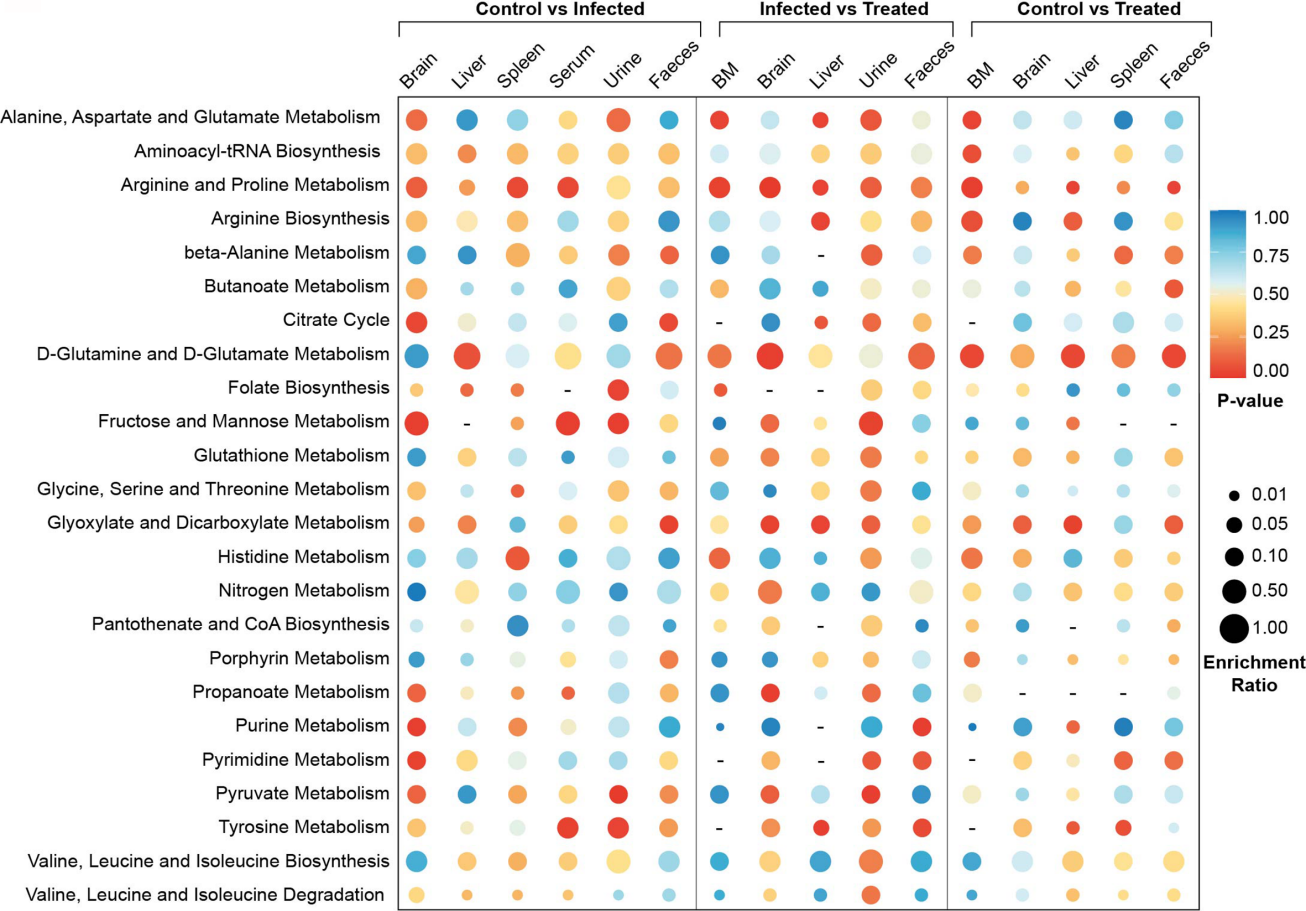
MSEA meta-analysis. Meta-analysis of significant pathways in different comparison groups—control *vs* infected, control *vs* treated and infected *vs* treated. MSEA is a cut-off-free method that evaluates the overall differences of two distributions based on Kolmogorov–Smirnov tests. *Mus musculus* metabolome library from KEGG was used for the MSEA. Enrichment ratio was calculated as the relative percentage of the empirical compound hits to the whole empirical pathway is denoted as the size of the circles. Significance of each of the pathways in a particular context is represented by their p-value, which is assigned as the various colors of the circles.

Metabolite set enrichment was done to identify and interpret patterns of metabolite concentration changes, which will further support the identified metabolites in absence of MS–MS quantification. Results showed maximum alterations in brain, urine and fecal samples during infection with respect to control (**Figure 4**). The bone marrow of infected samples showed almost no significant changes in the metabolite sets indicating a minimum alteration in their features during infection. In the case of infected *versus* treated serum and spleen samples, no significant changes were noticed in the metabolite sets which indicates VL-induced changes persist for a long time even after treatment in both sources. On the other hand, in control *versus* treated serum and urine samples, no significant enriched metabolite-sets were noticed showing that these biofluids quickly respond to treatment and normalize back to the control configuration. Most of the pathways showed less to no enrichment in the control *vs* treated category showing the progressive normalization process. The pathways that remain enriched in all tissue samples even after treatment are D-glutamine and D-glutamate metabolism, arginine biosynthesis, glyoxylate–dicarboxylate metabolism, valine–leucine–isoleucine biosynthesis and histidine metabolism. This indicates that a long time is taken by the host system to normalize these pathways even after minimizing the parasitic load.

Significantly identified metabolites of MSEA from the control *vs* infected group were considered for pathway enrichment by applying over representative analysis (ORA) as focussing on pathway enrichment during infection is crucial to identify the systemic changes that occur during VL progression.

**Figure 5:**
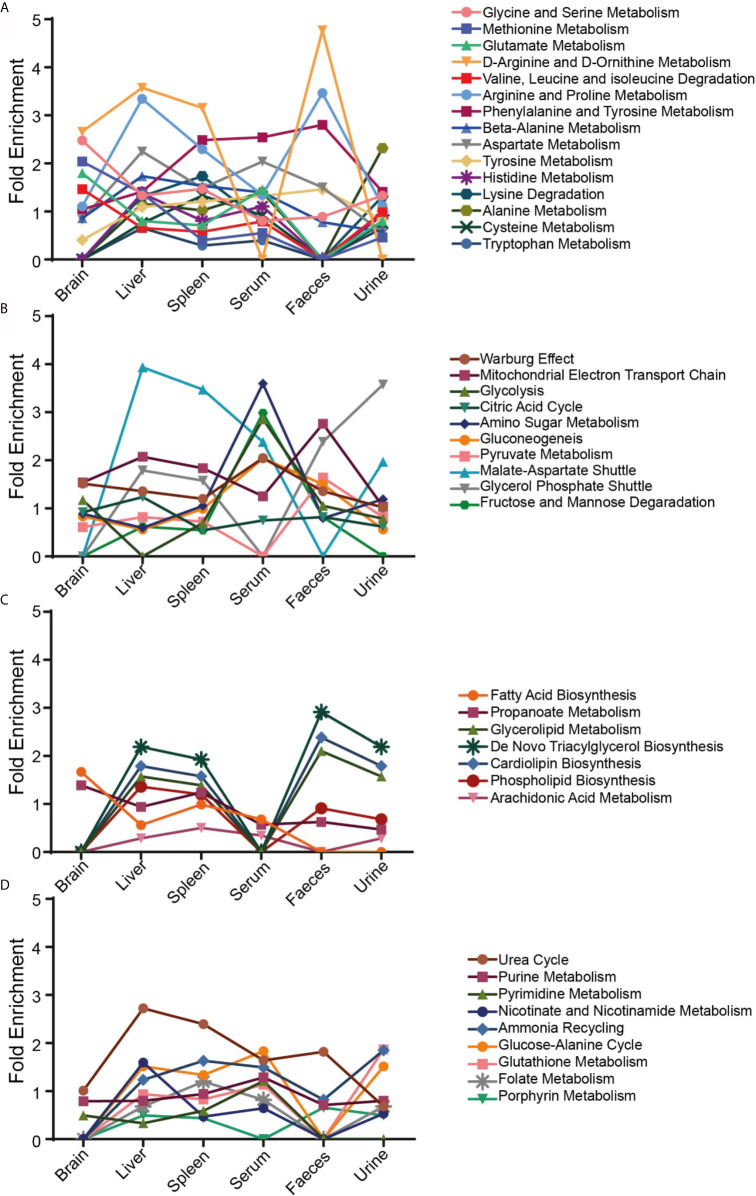
Fold enrichment with ORA. Pathway enrichment in different tissues and biofluids of the control *vs* infected samples was classified into four groups—**(A)** Amino acid metabolic pathways, **(B)** Energy related metabolic pathways, **(C)** Lipids and fatty acid metabolic pathways and **(D)** a miscellaneous group. ORA was implemented using the hypergeometric test to evaluate whether a particular metabolite set is represented more than expected by chance within the given compound list. It uses HMDB as a reference metabolome to calculate a background distribution to determine if the matched metabolite set is more enriched for a certain metabolite as compared to random chance.

The identified results were further filtered through HMDB (Human Metabolome database) to get a more vivid picture of modified pathways during VL. Metabolite abundance was found to be altered for multiple central metabolic pathways including amino acids, glycolysis, TCA, fatty acid synthesis and several other pathways (**Figure 5**). More than a few amino acid pathways like glycine, serine, methionine, arginine, proline, phenylalanine, tyrosine were found to be enriched in all tissue and biofluid samples. D-Arginine and D-Ornithine metabolism were highly altered in approximately all the tissue and biofluid samples except in serum, where no changes were perceived at all. Liver and spleen showed the maximum number of amino acid altered pathways (**Figure 5A**). Among energy-related pathways, the malate-aspartate shuttle was found to be most enriched in liver and spleen samples (**Figure 5B**). Glycolysis was also affected in all sample sources in a similar fashion, except in serum, where it was highly enriched (**Figure 5B**). *De-novo* triacylglycerol biosynthesis, cardiolipin biosynthesis and glycerolipid metabolic pathways followed a similar pattern for all samples. In serum and brain, no significant changes were detected in fatty-acid related metabolic pathways (**Figure 5C**). Urea cycle was found to be a highly altered pathway in liver, spleen and faeces (**Figure 5D**). Urine samples showed lesser changes in folate, porphyrin, glutathione, nicotinamide and urea cycle as compared to control.

### Metabolites Altered During Visceral Leishmaniasis Advancement

**Figure 6:**
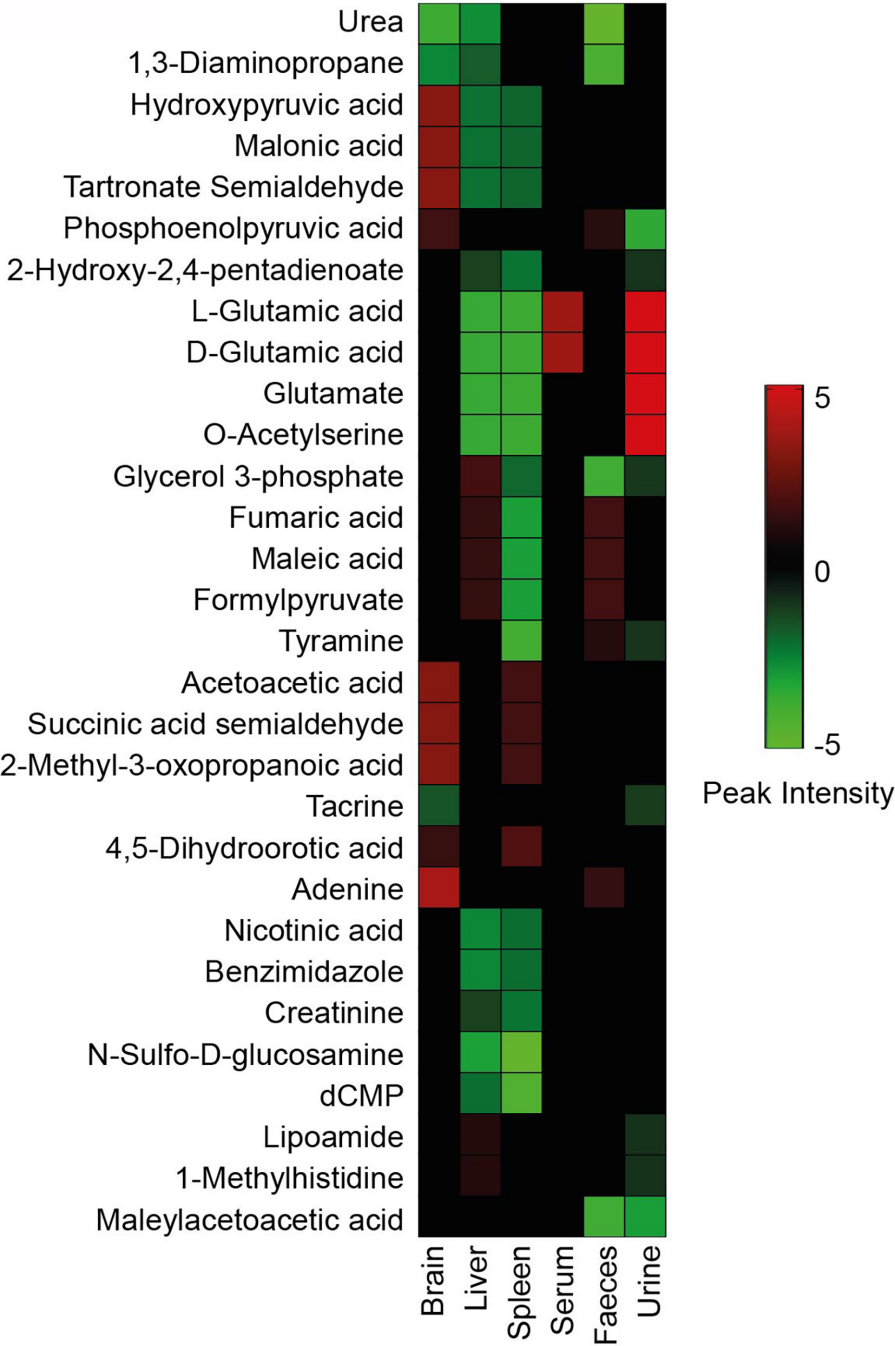
Metabolite heatmap. Significantly identified metabolites from the control *vs* infected group have been compared across different sources with respect to their normalized peak intensity. The color gradient ranging from red to green represents high to low peak intensity respectively.

Tissue-specific changes during *Leishmania* infection are an important aspect of comprehending the VL’s systemic effects. For this, several tissue-specific unique metabolites (**Tables 1**–**3**) and common metabolites (**Figure 6**) across systems were identified that were significantly altered in the infected samples as compared to control ones. Some of these metabolites remained altered even after treatment, showing the tendency of the host towards the disease environment. Therefore, this category of metabolites is very important in understanding the disease after-effects that need to be considered to address the VL systemic repercussions. The majority of metabolites decreased during infection as shown in **Figure 6**. Only a few metabolites like glutamic acid and O-acetylserine showed an elevated pattern in serum and feces of infected BALB/c mice. Other molecules that were in higher amounts in infected mice’s brain samples are hydroxypyruvic acid, malonic acid, tartronate semialdehyde, acetoacetic acid, succinic acid semialdehyde and methyl-3-oxopropanoic acid. Liver and spleen mostly showed declining features after infection (**Figure 6**). The highest number of unique metabolite alterations were observed in the brain (**Table 1**), followed by spleen (**Table 2**) and urine (**Table 3**). Very few metabolites were identified in liver (**Table 1**), serum (**Table 2**) and feces (**Table 3**) that were specifically unique to these tissues. No unique modifications were noticed in bone marrow samples. A higher rate of downregulation in metabolites of the infected samples point towards the suppressive behaviour of VL.

**Table 1:** Unique metabolites identified in Brain and Liver (Control *vs* infected).

Metabolites unique to brain	log2(FC)		-log10(p)
**Orotic acid**	3.1495		2.8825
**Indole**	2.035		2.5738
**Benzimidazole**	2.0933		2.3077
**L-Threonine**	2.0933		2.3077
**L-Homoserine**	2.0933		2.3077
**Gamma-Aminobutyric acid**	2.0933		2.3077
**D-Threonine**	2.0933		2.3077
**Dimethylglycine**	2.0933		2.3077
**(R)-b-aminoisobutyric acid**	2.0933		2.3077
**Aminoacetone**	2.0933		2.3077
**Acetoxime**	2.0933		2.3077
**2-Methylserine**	2.0933		2.3077
**Xanthine**	3.2915		2.0985
**N-(6-Aminohexanoyl)-6-aminohexanoate**	−1.472		1.8455
**2,5-Dihydroxypyridine**	3.436		1.6846
**Dihydroxyfumaric acid**	1.4068		1.6846
**D-Erythrose 4-phosphate**	1.5557		1.6117
**Succinic acid**	2.0513		1.5444
**2-Dehydro-3-deoxy-L-arabinonate**	2.0513		1.5444
**Citramalic acid**	2.0513		1.5444
**D-2-Hydroxyglutaric acid**	2.0513		1.5444
**L-Arabinono-1,4-lactone**	2.0513		1.5444
**Methylmalonic acid**	2.0513		1.5444
**Metabolites unique to liver**			
**Caproic acid**	−1.1011		4.8558
**Deazaflavin**	−1.3104		3.617
**Choline**	8.9752		1.6642
**Tropic Acid**	7.2364		1.601

**Table 2:** Unique metabolites identified in Spleen and Serum (Control *vs* Infected).

Metabolites unique to spleen	log2(FC)	−log10(p)
**Proline**	2.1046	6.5921
**Dihydroxyacetone**	2.1046	6.5921
**5-Aminolevulinic acid**	2.1046	6.5921
**Hydroxypropionic acid**	2.1046	6.5921
**N-Acetyl-beta-Alanine**	2.1046	6.5921
**5-Amino-2-oxopentanoic acid**	2.1046	6.5921
**Hydroxyproline**	2.1046	6.5921
**Lactic Acid**	2.1046	6.5921
**Glyceraldehyde**	2.1046	6.5921
**3-Mercaptopyruvic acid**	1.8048	5.4947
**Dihydropteroate**	1.7317	5.0693
**Delta1-Piperideine-6-L-carboxylate**	1.6456	4.7189
**Aminoadipic acid**	1.6456	4.7189
**beta-Alanopine**	1.6456	4.7189
**Sulfoacetaldehyde**	1.4976	4.1812
**Pyrroloquinoline quinone**	1.4038	3.5118
**Dihydrolipoic acid**	1.2324	2.9361
**Metabolites unique to serum**		
**L-4-Hydroxyglutamate semialdehyde**	3.7024	1.7297
**5-Thymidylic acid**	−1.2675	1.1772
**Caprylic acid**	−1.1952	1.9015
**(S)-3-Methyl-2-oxopentanoic acid**	−5.1114	2.1965

**Table 3:** Unique metabolites identified in Urine and Feces (Control *vs* Infected).

Metabolite	log2(FC)	−log10(p)
**Isopentenyl pyrophosphate**	−1.3312	1.6146
**Dethiobiotin**	−1.3528	1.6146
**Indanone**	−1.1113	1.6146
**Pyridoxal**	−1.2638	1.6146
**3-Isopropylcatechol**	−1.2133	1.6146
**Glycine**	−1.135	1.6146
**Inosine**	−1.1796	1.6146
**L-Fuculose 1-phosphate**	−1.1796	1.6146
**L-Rhamnulose 1-phosphate**	−1.1796	1.6146
**(R)-3,3-Dimethylmalate**	2.6593	1.6146
**3-Ethylmalate**	2.6593	1.6146
**Guanidoacetic acid**	2.6593	1.6146
**4-Aminobutyraldehyde**	−1.0412	1.5221
**3’,5’-Cyclic IMP**	−1.0756	1.5197
**N-Acetyl-L-glutamate 5-semialdehyde**	−1.0327	1.4573
**3-Propylmalate**	−1.0255	1.5197
**Metabolites unique to Faeces**		
**Asparagine**	1.1315	1.5469
**N-Carbamoylsarcosine**	1.1315	1.5469
**Glycyl-glycine**	1.1315	1.5469
**3-Oxalomalate**	1.4637	1.5306
**dIDP**	1.4637	1.5306

### Prognostic/Diagnostic Marker Identification

To understand the disease progression and modulation in host, we looked for the tissue-specific changes while witnessing the control, infected and treated samples altogether. For this, pattern hunter analysis was done using the spearman rank correlation algorithm, which was set to find a 1-2-1 pattern. This led to the identification of several metabolites, that were specifically modified in infected samples.

### Tissues

**Figure 7:**
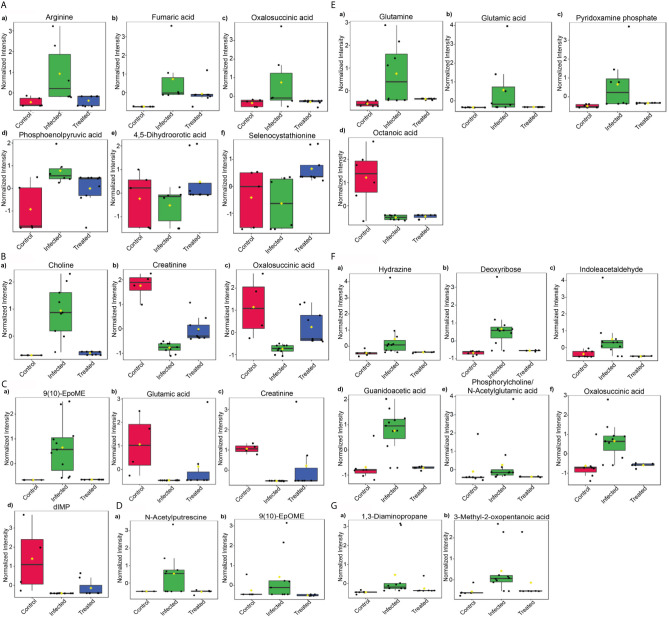
Potential biomarkers. Pattern search using the predefined pattern 1-2-1 (control-infected-treated) based on Spearman rank correlation was used to identify potential biomarkers in tissues—**(A)** Brain, **(B)** Liver, **(C)** Spleen, **(D)** Bone marrow and biofluids—**(E)** Serum, **(F)** Urine, and **(G)** Feces on the basis of their normalized intensity.

After an analysis of brain samples with pattern hunter, arginine, fumaric acid, oxalosuccininc acid and phosphopyruvic acid were found to be upregulated specifically during infection, whereas 4,5-Dihydroorotic acid and selenocystathionine significantly declined during infection. These metabolites were found to regain a pattern similar to that of control samples after treatment (**Figure 7A**). In liver samples, choline dramatically increased during infection whereas creatinine and oxalosuccininc acid decreased during the duration of infection. These metabolites had comparable normalized intensity in control and treated samples (**Figure 7B**). In splenic samples, 9(10)-EpoME was the only metabolite that showed increment during infection while others like glutamic acid, creatinine and dIMP decreased during the infection (**Figure 7C**). During the course of infection, N-acetylputrescine and 9(10)-EpOME were the only ones found to be upregulated in bone marrow samples that returned to control configuration after treatment (**Figure 7D**).

### Biofluids

The major objective behind profiling biofluids was to find leads for possible diagnostic markers. Following this direction, several molecules were identified that were either serum, urine or feces specific. In serum samples, a marked increase in the intensity of glutamine, glutamic acid and pyridoxamine phosphate was noticed during infection whereas octanoic acid decreased during infection. Treated samples followed a pattern similar to that of control samples, with the exception of octanoic acid, which remained downregulated even after declined parasitic burden (**Figure 7E**). In urine, the maximum number of metabolites identified were modulated specifically during infection. Among them, hydrazine, deoxyribose, indoleacetaldehyde, guanidoacetic acid, oxalosuccinic acid, thymidine, nicotinate D-ribonucleotide and xanthosine showed elevation during infection in urine samples only. Although increasing, phosphorylcholine and N-acetylglutamic acid showed lower intensity during infection as compared to control and treated samples. 2,3-Diphosphoglyceric acid showed a significant decrease in infected and control samples (**Figure 7F**). In fecal samples, only two metabolites i.e. 1,3-diaminopropane and 3-methyl-2-oxopentanoic acid were uniquely identified, exhibiting increased intensity during infection (**Figure 7G**).

## Discussion

Deciphering host manipulation during pathogenic diseases is one of the emerging approaches to untangle the complexity of infection mechanisms and to identify prognostic markers of biological activity (Somvanshi and Venkatesh, 2014; Khan et al., 2019). *Leishmania donovani* (Ld), a causative agent of visceral leishmaniasis (VL) is well known for its host alteration in several aspects (Olivier et al., 2005; Pandey et al., 2016). A lot of studies have described host reconfiguration upon *Leishmania* infection, through gene expression and translational studies but it may not reflect overall biological activities and metabolic changes which portray the infection scenario (Rabhi et al., 2012; Veras et al., 2018; Chaparro et al., 2020). Earlier studies have focused on cellular modifications *in vitro*, but it is still lagging in the *in vivo* assessment so as to address the detailed trend of composition and heterogeneity of host during VL (Lamour et al., 2012). Metabolic signatures have recently been identified in the case of several diseases and are also very helpful in defining vital processes of the body like ageing and nutrient-dependent changes of body (Wuolikainen et al., 2011; Fan et al., 2018; Chak et al., 2019). Here, we studied BALB/c mice’s tissue and biofluid specific changes during *L. donovani* infection through untargeted metabolomics resulting in a complete picture of cumulative host modifications during VL. Untargated metabolomics is preferred over targeted one, to identify thousands of small molecules of unknown chemical properties; thus, helping in characterizing molecular damage patterns by following changes in metabolite diversity (Dias et al., 2016; Gertsman and Barshop, 2018). Liver, spleen and bone marrow are central in VL agonized parts, and hence were selected for profiling (Kaye et al., 2004; Bankoti and Stäger, 2012). The motive behind choosing brain under this category is that brain inflammation was also reported in VL, and results will help in identifying the missing link between the neuroinflammation present in VL and related mental health problems (Alemayehu et al., 2017; Melo et al., 2017). Biofluid (serum, urine, feces) profiling was chosen in the study for biomarker/prognostic marker identification. Moreover, they can be obtained through less invasive methods and therefore are best suitable under this category. Another important aspect of this study is tissue-specific profiling, which has an advantage in addressing questions like how *Leishmania* infection results in a systemic disorder (Costa et al., 2010). This approach will also help to comprehend the disease pathogenesis and its progression. Further outputs are the identification of prognostic biomarkers and therapeutic targets.

Results of the profiling clearly indicate a differential tone of metabolic pathways between control and Ld infected mice’s tissue and biofluid samples. A variety of differential metabolites have been identified; which includes a range of amino acids, saccharides, energy-related molecules, etcetera. Reviewing amino acid modulation in the host is also important from the point of view that intracellular amastigotes can uptake essential amino acids from the phagolysosomes for their survival (Burchmore and Barrett, 2001; Olivier et al., 2005; Saunders and McConville, 2020). This is one of the most important mechanisms to attenuate the host’s immune response by pathogens, through depleting their amino acid pool, which is central to the immune mechanism (Grohmann and Bronte, 2010; McGaha et al., 2012; Zhang and Rubin, 2013).

Pathway enrichment and further meta-analysis showed that some of the metabolic pathways were altered in all the samples during Ld infection, helping us narrow down the most significant pathways that have an overall systemic effect; these include—phenylalanine, tyrosine, D-Arginine, D-Ornithine, malate-aspartate, urea, etcetera (**Figures 4**, **5**) metabolic pathways. Also, tissue-specific pathway changes were noticed, indicating their possible role in affecting the specific functionality of the tissue during VL. It is well known that utilization of arginine by both, host and pathogen represents a metabolic bottleneck which is decisive in shaping the fate of a pathogenic infection. For e.g. Arginase (important enzyme of parasite) competes with iNOS (important defense of macrophage to generate NO) for arginine; as many pathogens including *Leishmania* exploit this to block NO production by increasing expression of arginase, to limit arginine availability for metabolism *via* iNOS (Ren et al., 2018). Also, several pathogens like *Plasmodium yoelii* infection alters concentrations of several amino acids in plasma of infected mice, like valine, leucine, tyrosine, phenylalanine, EOHNH2, histidine, proline, aspartate, glutamate, alanine, etcetera (Saiki et al., 2013). During infection, host cell redirects its metabolic fluxes towards the strengthening of its defense whereas the pathogen modulates host’s micro-environment to derive its nutritional requirement as well as securing its survival. The overall changes in host portray both of these aspect.

The pathways that quickly responded to the treatment are working like switches for VL and could be further targeted for prognosis marker identification for e.g.—arginine, proline, beta-alanine, folate biosynthesis, porphyrin, pyruvate, etcetera in metabolic pathways (**Figure 4**). The pathways that were altered after treatment but did not respond completely to the treatment for e.g.—D-glutamine, D-glutamate, glyoxylate, dicarboxylate, citrate cycle, etcetera, are the ones that could be further looked into for their direct role in affecting the host’s physiology for a prolonged time and revealing their activity in maintaining the disease complexity even after the successful completion of treatment (**Figure 4**). Although glucose is an important metabolite, and the main fuel for a large number of cells in the body, but cells like lymphocytes, neutrophils, and macrophages, consume glutamine at high rates under catabolic conditions; such as sepsis, recovery from burns or surgery, etcetera (Cruzat et al., 2018). Glutamine is an important precursor for the generation of other metabolites, such as amino acids (glutamate), TCA components (α-ketoglutarate), and nucleotides (AMP, purines, and pyrimidines), along with the activation of the chaperone function (mediated by HSP response) and antioxidant defence (mediated by glutathione, GSH) (Cruzat et al., 2018). Therefore, glutamine alteration seeks further studies.

Several common metabolite-sets that were up/down-regulated during the Ld infection, include an eclectic collection of molecules from TCA, glycolysis, to fatty acids, amino acids, purine and pyrimidine metabolic pathways, etcetera; indicating their role in disease prognosis and advancement. Metabolites that quickly responded to the treatment, could be further explored for prognosis/diagnosis development. Several metabolites identified in this category have a healing effect on the system, like arginine (identified in the brain samples) reduces brain edema formation and improves cortical blood flow (Bemana and Nagao, 1999). Fumaric acid is known to exert neuroprotective effects in neuroinflammation *via* activation of the Nrf2 antioxidant pathway (Linker et al., 2011). It is also a well-known antibacterial agent. A decline in orotic acid (identified in brain) helps balance the aciduria and academia in the system (Löffler et al., 2015). Liver choline is known to have a healing effect on the liver by removing extra fat (Cohen et al., 2011). Decreased succinate in liver (as identified in liver and spleen) has a shielding effect on liver damage (Correa et al., 2007). EpOME (identified in bone marrow and spleen) is known to be generated by neutrophils during the oxidative burst (Hildreth et al., 2020).

Several other identified metabolites have known systemic suppressive effects; like oxalosuccinic acid is a metabolic signature of hypoxia (Connors et al., 2019). Low creatinine is a sign of poor liver function (Beben and Rifkin, 2015). A marked decline observed in metabolites like glutamic acid, creatinine and dIMP in spleen needs to be further studied for its functional aspects. Elevated glutamine and glutamic acid in serum are known to be associated with kidney dysfunction (Fadel et al., 2014). Increased pyridoxamine phosphate has a protective response in serum (Rej et al., 1973). Urine sample yielded eight metabolites that could be further explored for their potential in diagnostics. Other identified metabolites could be further explored for their possible tissue-specific role in VL. Identified metabolites and pathways could be further explored for host targeted therapy. This study becomes crucial in the current scenario, where drug resistance and evolution of mutated pathogen hampers the therapeutic approaches (Wise, 2021). Host targeted therapy helps to strengthen the host in way so as the current therapeutic strategy remains sufficient for dealing with the competitive evolution. Our identified metabolic pathways and metabolites could be further explored for their potential in VL prognosis and hence, their role in VL pathogenesis. Tissue/biofluid specific approach of the present study is very advantageous to understand the systemic effect of VL and further revealing the path of easy diagnostics. The output of the current study can also be beneficial in other microbial and health complications.

## Data Availability Statement

The raw data supporting the conclusions of this article will be made available by the authors, without undue reservation.

## Ethics Statement

The animal study was reviewed and approved by Animal Ethics Committee of the National Institute of Immunology, New Delhi (IAEC/AQ/2019/185, serial no. IAEC#454/17).

## Author Contributions

SD, TS, and CS designed the experiments. SD and TS performed, analyzed the data, and wrote the article. All authors revised the manuscript. All authors contributed to the article and approved the submitted version.

## Funding

SD was a recipient of DST INSPIRE faculty award (grant no. DST/INSPIRE/04/2015/002785) by DST (Department of Science and Technology), India. TS (JRF) was supported by a research grant of DST INSPIRE faculty award. CS is Professor of Eminence at the National Institute of Immunology and funded by Institutional Core funding and recipient of JC Bose fellowship from DST. The work was financially supported by DST Inspire Faculty award by DST, India.

## Conflict of Interest

The authors declare that the research was conducted in the absence of any commercial or financial relationships that could be construed as a potential conflict of interest.

## Publisher’s Note

All claims expressed in this article are solely those of the authors and do not necessarily represent those of their affiliated organizations, or those of the publisher, the editors and the reviewers. Any product that may be evaluated in this article, or claim that may be made by its manufacturer, is not guaranteed or endorsed by the publisher.

## Abbreviations

VL, visceral leishmaniasis; Ld, *Leishmania donovani*; GC-MS, gas chromatography- mass spectrometry; TIC, total ion chromatogram; Ld, *Leishmania donovani*; MSEA, metabolite set enrichment analysis; ORA, over representation analysis; PCA, principal component analysis; PLS-DA, partial least-squares discriminant analysis.
