# Supplementary material for: Tissue/Biofluid Specific Molecular Cartography of Leishmania donovani Infected BALB/c Mice: Deciphering Systemic Reprogramming
Source: Front Cell Infect Microbiol. 2021 Jul 29;11:694470. doi: 10.3389/fcimb.2021.694470 (PMC8358651; doi:10.3389/fcimb.2021.694470)
Supplement: Supplementary file 1 [file Image_1.pdf]

Figure S1

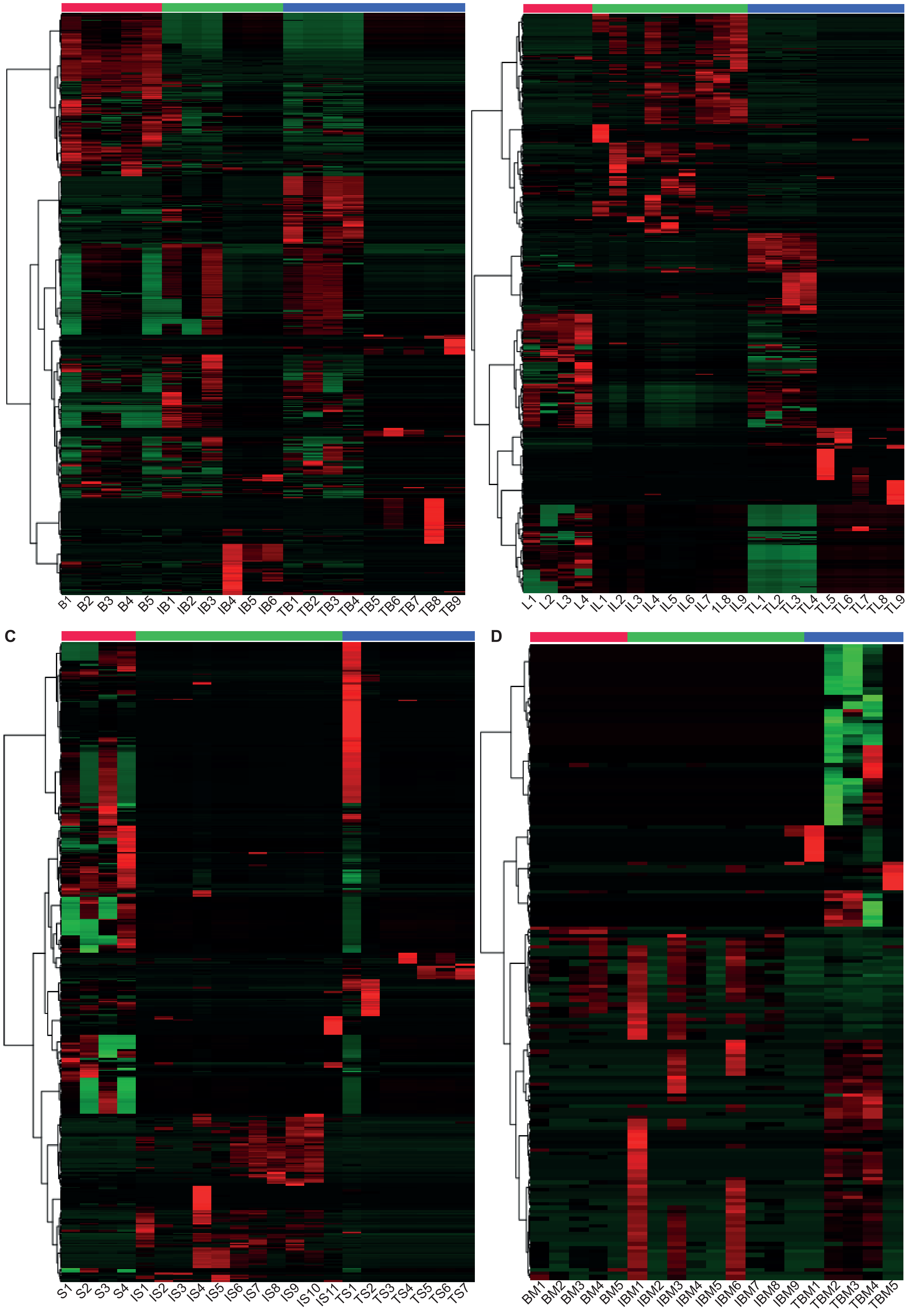
B1 B2 B3 B4 B5 IB1 IB2 IB3 IB4 IB5 IB6 TB1 TB2 TB3 TB4 TB5 TB6 TB7 TB8 TB9
L1 L2 L3 L4 IL1 IL2 IL3 IL4 IL5 IL6 IL7 IL8 IL9 TL1 TL2 TL3 TL4 TL5 TL6 TL7 TL8 TL9
C
S1 S2 S3 S4 IS1 IS2 IS3 IS4 IS5 IS6 IS7 IS8 IS9 IS10 IS11 TS1 TS2 TS3 TS4 TS5 TS6 TS7
D
BM1 BM2 BM3 BM4 BM5 IBM1 IBM2 IBM3 IBM4 IBM5 IBM6 IBM7 IBM8 IBM9 TBM1 TBM2 TBM3 TBM4 TBM5

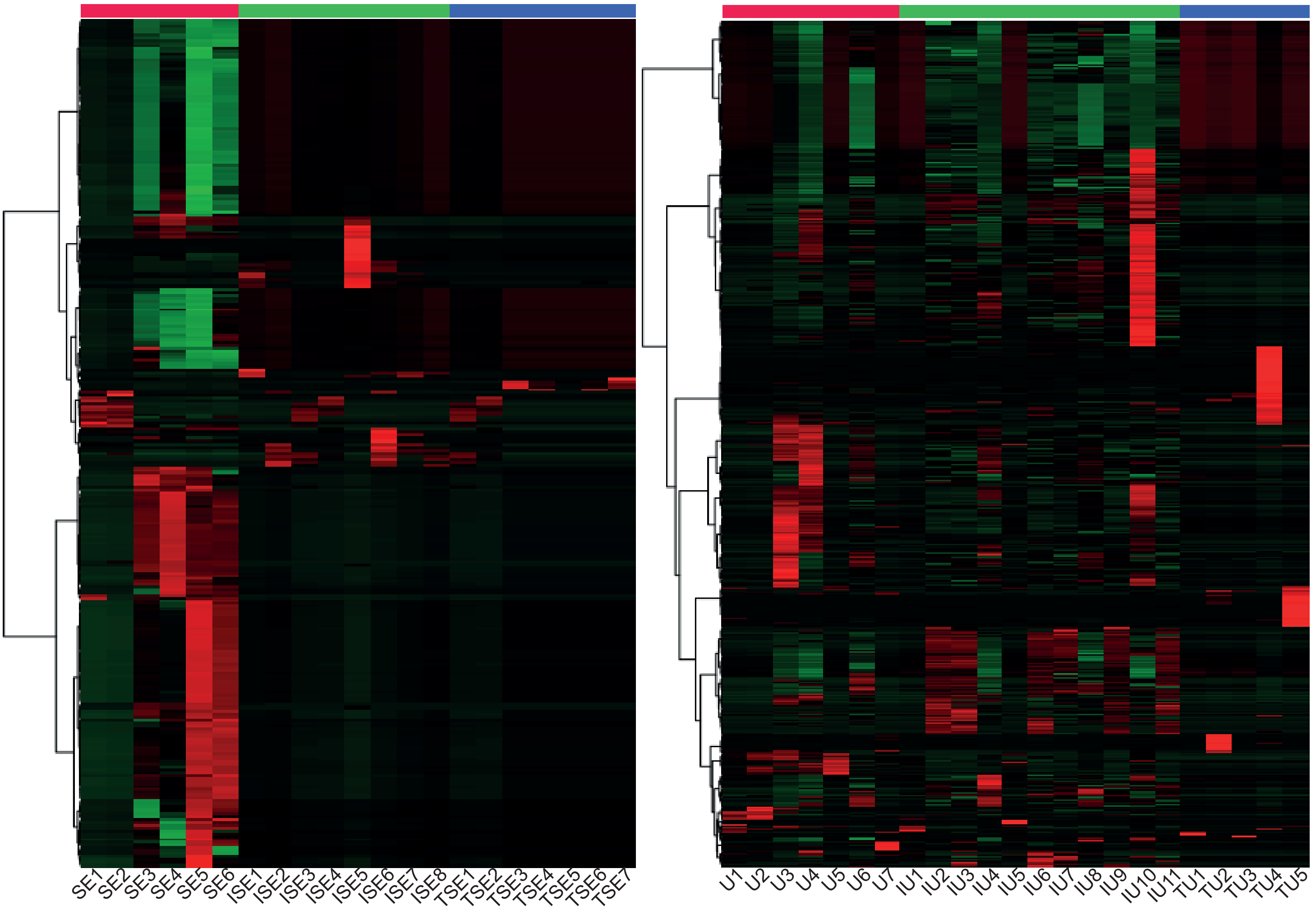

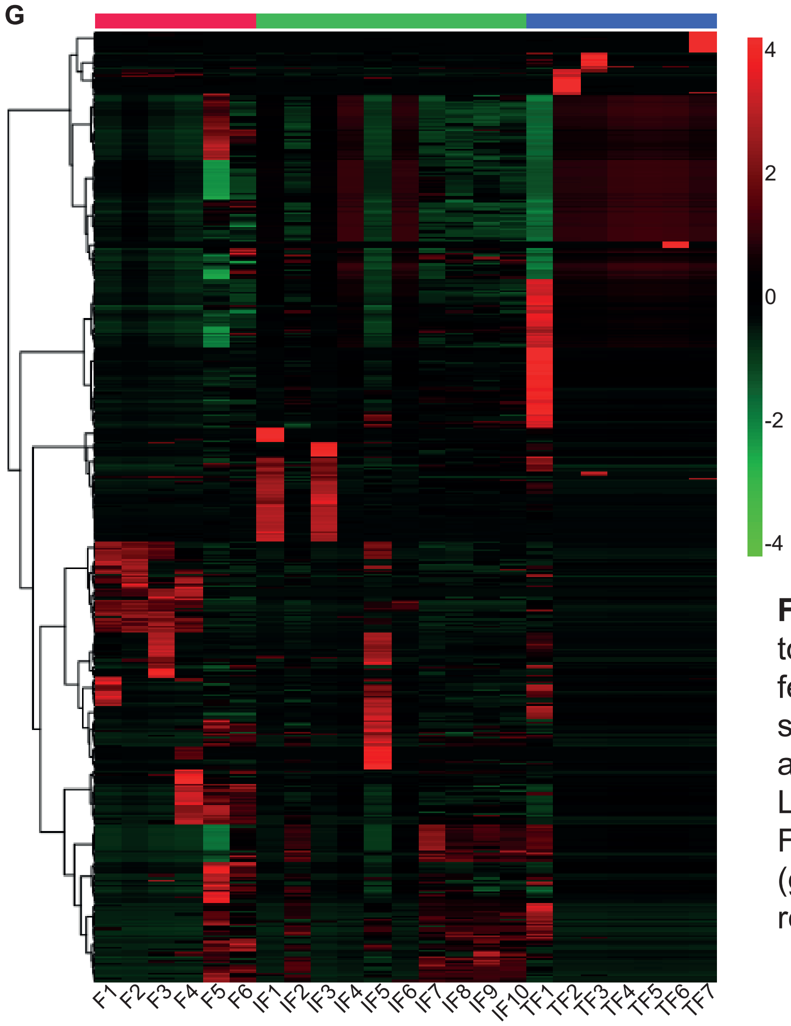

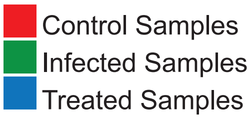

Figure S1. Heatmap. Hierarchical clustering to show clear separation of the identified feature profiles of control, infected and treated samples using Euclidean distance measure and ward.D clustering algorithm in A) Brain, B) Liver, C) Spleen, D) Bone marrow, E) Serum, F) Urine and G) Faeces. Warm (red) to cold (green) color denotes upregulated and down-regulated features respectively.

Figure S2

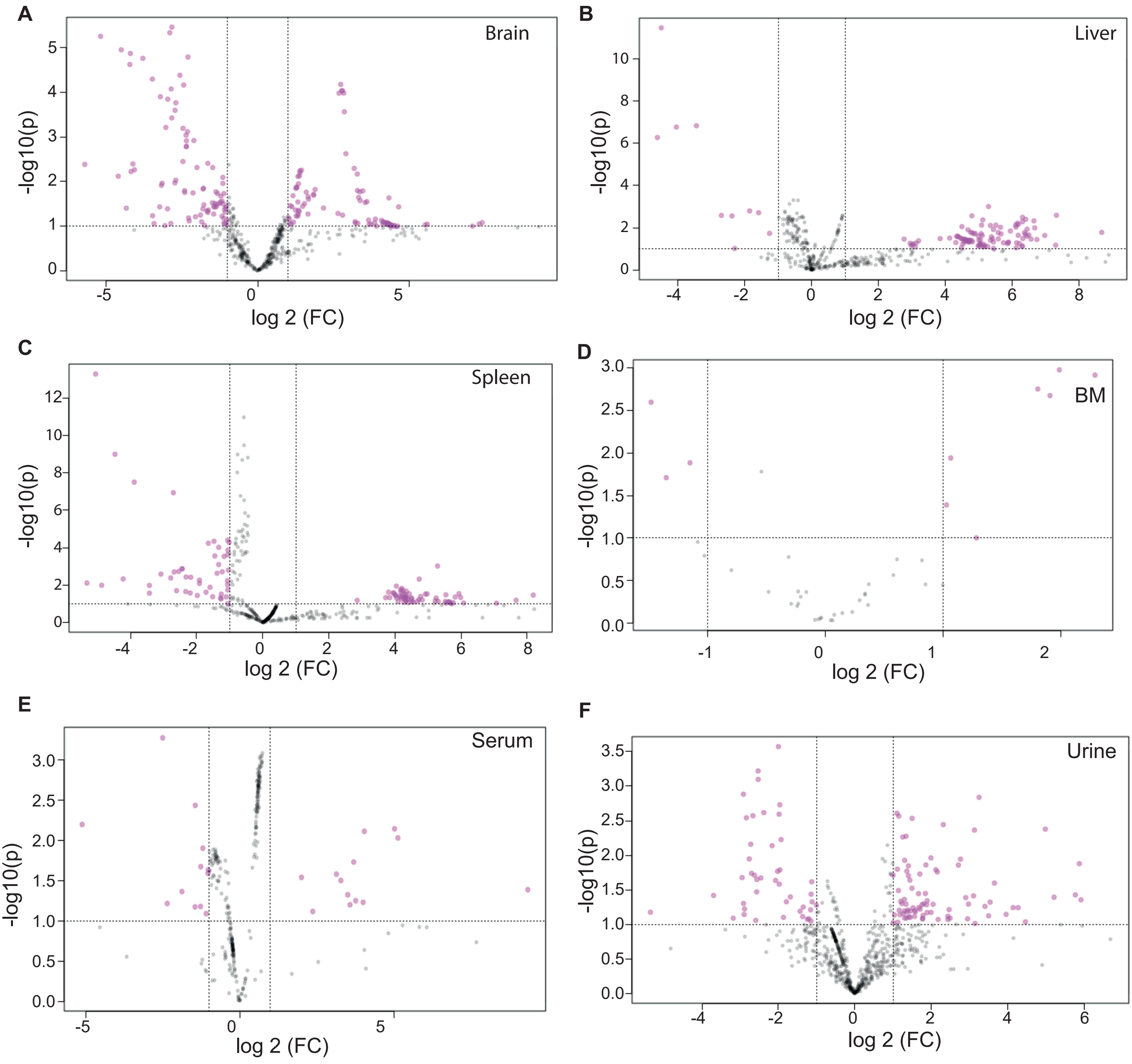

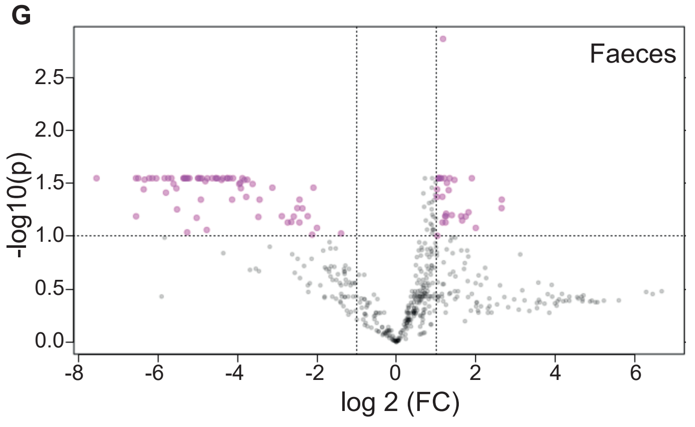

Figure S2. Volcano plot (control vs infected group). Volcano Plot is a variant of scatter plot that shows statistical significance (P value) versus Magnitude of change (fold change) of different tissues and biofluids. Important features selected by volcano plot with fold change threshold is 2 (x axis) and t-tests threshold is (y-axis) 0.1. The red circles represent features above the threshold. Note both fold changes and p values are log transformed. The further its position away from the (0,0), the more significant the feature is. A) Brain B) Liver C) Spleen D) Bone marrow E) Serum F) Urine G) Faeces.

Figure S3

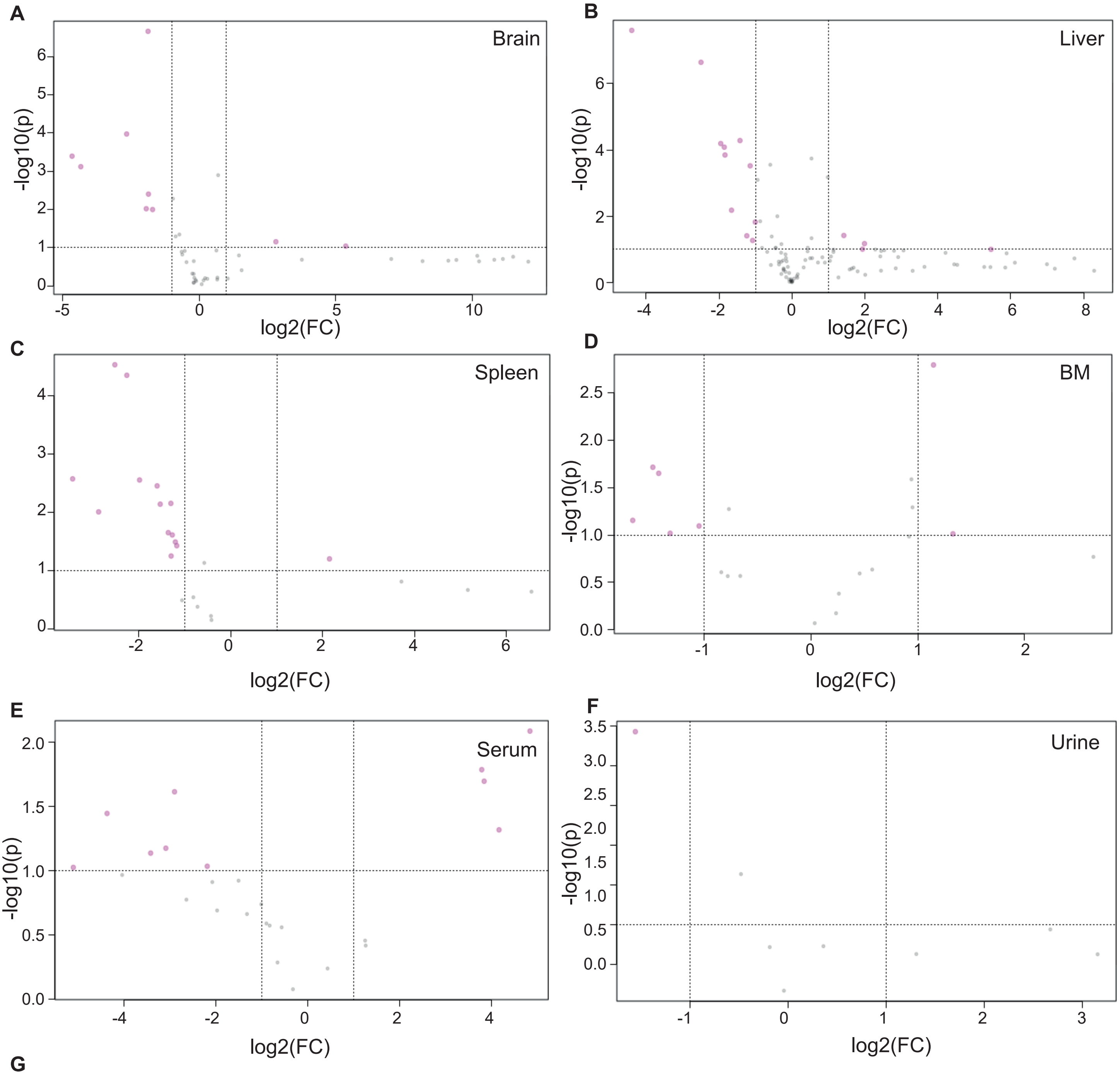

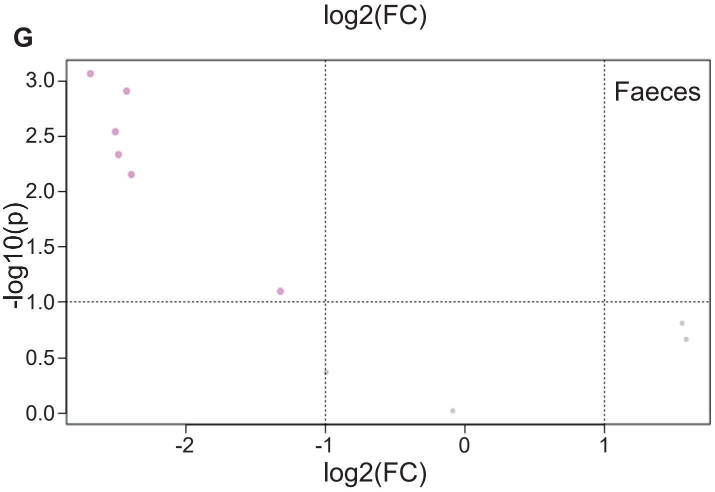

Figure S3. Volcano plot (control vs treated group). Important features selected by volcano plot with fold change threshold is 2 (x axis) and t-tests threshold is (y-axis) 0.1. The red circles represent features above the threshold. Note both fold changes and p values are log transformed. The further its position away from the (0,0), the more significant the feature is. A) Brain B) Liver C) Spleen D) Bone marrow E) Serum F) Urine G) Faeces.
